# CCL21/CCR7 Axis Contributed to CD133^+^ Pancreatic Cancer Stem-Like Cell Metastasis via EMT and Erk/NF-κB Pathway

**DOI:** 10.1371/journal.pone.0158529

**Published:** 2016-08-09

**Authors:** Lirong Zhang, Dongqing Wang, Yumei Li, Yanfang Liu, Xiaodong Xie, Yingying Wu, Yuepeng Zhou, Jing Ren, Jianxin Zhang, Haitao Zhu, Zhaoliang Su

**Affiliations:** 1 Department of Radiology, The Affiliated Hospital of Jiangsu University, Zhenjiang, 212001, China; 2 Department of Immunology, Jiangsu University, Zhenjiang, 212013, China; 3 Department of Central laboratory, The First People’s Hospital of Zhenjiang, Zhenjiang, 212001, China; Universitat Witten/Herdecke, GERMANY

## Abstract

**Background:**

Tumor metastasis is driven by malignant cells and stromal cell components of the tumor microenvironment. Cancer stem cells (CSCs) are thought to be responsible for metastasis by altering the tumor microenvironment. Epithelial-mesenchymal transition (EMT) processes contribute to specific stages of the metastatic cascade, promoted by cytokines and chemokines secreted by stromal cell components in the tumor microenvironment. C-C chemokine receptor 7 (CCR7) interacts with its ligand, chemokine ligand 21(CCL21), to mediate metastasis in some cancer cells lines. This study investigated the role of CCL21/CCR7 in promoting EMT and metastasis of cluster of differentiation 133^+^ (CD133^+^) pancreatic cancer stem-like cells.

**Methods:**

Panc-1, AsPC-1, and MIA PaCa-2 pancreatic cancer cells were selected because of their aggressive invasive potentials. CCR7 expression levels were examined in total, CD133^+^ and CD133^−^ cell fractions by Immunofluorescence analysis and real time-quantitative polymerase chain reaction (RT-qPCR). The role of CCL21/CCR7 in mediating metastasis and survival of CD133^+^ pancreatic cancer stem-like cells was detected by Transwell assays and flow cytometry, respectively. EMT and lymph node metastasis related markers (E-cadherin, N- cadherin, LYVE-1) were analyzed by western blot. CCR7 expression levels were analyzed by immunohistochemical staining and RT-qPCR in resected tumor tissues, metastatic lymph nodes, normal lymph nodes and adjacent normal tissues from patients with pancreatic carcinoma.

**Results:**

CCR7 expression was significantly increased in CD133^+^ pancreatic cancer stem-like cells, resected pancreatic cancer tissues, and metastatic lymph nodes, compared with CD133^−^ cancer cells, adjacent normal tissues and normal lymph nodes, respectively. CCL21/CCR7 promoted metastasis and survival of CD133^+^ pancreatic cancer stem-like cells and regulated CD133^+^ pancreatic cancer stem-like cells metastasis by modulating EMT and Erk/NF-κB pathway.

**Conclusions:**

These results indicate a specific role for CCL21/CCR7 in promoting EMT and metastasis in CD133^+^ pancreatic cancer stem-like cells. Furthermore the data also indicated the potential importance of developing therapeutic strategies targeting cancer stem-like cells and CCL21/CCR7 for reducing metastasis.

## Introduction

Tumor metastasis is responsible for >90% of pancreatic cancer mortality. Extensive research has determined that metastasis involves several steps, including invasion of adjacent tissues, generation of circulating tumor cells (CTCs), intravasation in blood or lymphatic vessels, survival in the vasculature, and extravasation and growth at secondary sites [1, 2]. Each stage of metastasis requires close collaboration between cancer cells and various stromal cell components, which secrete cytokines, growth factors, and proteases that remodel the tumor microenvironment [3–5].Whereas, it remains unclear how the tumor-associated factors influence tumor metastasis.

Accumulating evidence suggests that malignant cells on the tumor border can develop invasive, mesenchymal characteristics that facilitate tumor detachment and acquisition of a migratory phenotype [6–8]. EMT is a complex series of morphological changes culminating in the loss of epithelial characteristics and the acquisition of a motile mesenchymal phenotype. In the context of cancer, EMT facilitates the dissemination of cancer cells and endows them with properties essential for metastasis, including stemness, invasiveness, and the ability to survive in the circulation and seed at secondary site. The regulation of EMT is likely to depend on non-tumor cells and a variety of growth factors, cytokines, and chemokines in the tumor microenvironment [9, 10]. However, the mechanisms whereby these tumor-associated factors influence EMT are unclear. Seminal work demonstrated that EMT generates mesenchymal-like cells with properties associated with CSCs [11], and the abilities of epithelial cancer cells to undergo EMT and acquire CSC properties are believed to play critical roles in metastasis.

Chemokines are low-molecular weight, pro-inflammatory cytokines described as important mediators of tumor metastasis, proliferation, and apoptosis [12, 13]. The CC chemokine receptor7 (CCR7) is predominantly expressed in various subsets of T lymphocytes and activated dendritic cells (DC), and interaction with its ligand chemokine ligand 21 (CCL21) recruits these cell populations to the lymph nodes. CCL21 has been shown to be responsible for mediating metastasis, especially lymph node metastasis, in certain cancer cells lines [14–16].

Pancreatic carcinoma is the most common cause of cancer-related mortality worldwide. The primary reason for the poor outcome in pancreatic carcinoma patients is the presence of extensive local tumor invasion and frequent spread to metastatic sites, particularly the lymph nodes[17].

The current study investigated the ability of exogenous cytokine signaling from the tumor microenvironment to promote pancreatic CSC metastasis and survival through activation of EMT. The results suggest that targeting EMT can be disrupted by inhibiting the generation of soluble factors by tumor-associated stromal cells, which may represent an effective strategy for inhibiting tumor progression and metastasis, leading to improved patient outcomes.

## Results

### CCR7 expression in CD133^+^ pancreatic cancer stem-like cells

**Fig 1:**
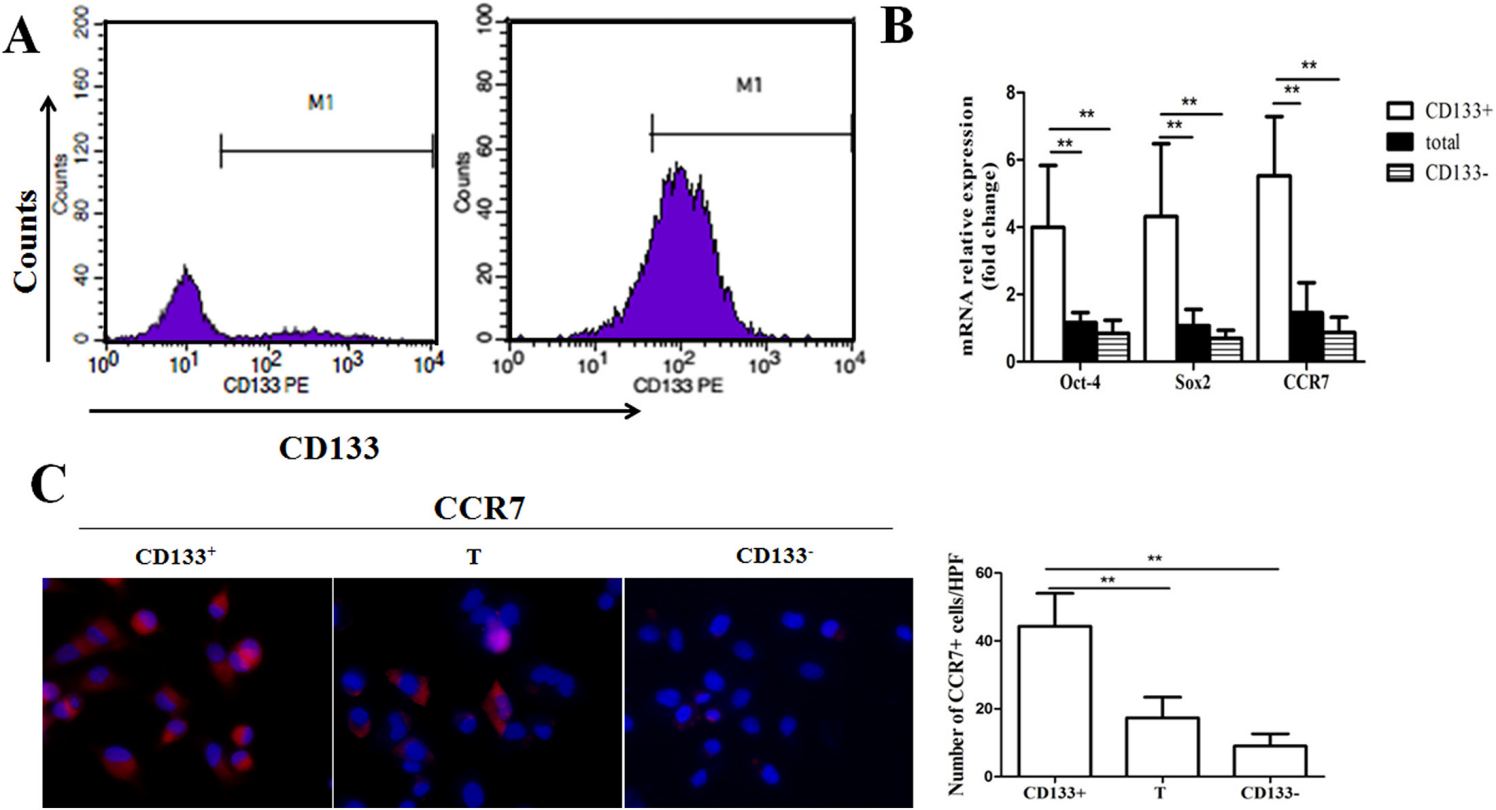
Expression levels of stemness related markers and CCR7 in CD133^+^ pancreatic cancer stem-like cells. (A) Sorted CD133^+^ cancer cells and the total cells were cultured in the serum-free DMEM-F12 medium for 72h. The percentage of CD133^+^ in the total cell lines and sorted CD133^+^ were tested by FACS. The results displayed that purity of the CD133^+^ were 14.73% and 91.84%, respectively. (B) Oct-4, Sox-2, and CCR7 mRNA levels in total pancreatic cancer cells and in CD133^+^ and CD133^−^ cell fractions detected by RT-qPCR. Data were normalized to β-actin levels. Experiments were repeated three times with similar results. (C) CCR7 expression levels in total pancreatic cancer cells and in CD133^+^ and CD133^−^ cell fractions were detected by immunofluorescence staining (200×),(*P<0.05, **P<0.01, ***P<0.001).

CD133^+^ and CD133^−^ cells were sorted from total Panc-1 cell line by FACS. The sorted CD133^+^ and the total cells were cultured in the serum-free DMEM-F12 medium. After 3 days, the purity of CD133^+^ was 91.84% and 14.73%, respectively (Fig 1A).To confirm that CD133^+^ cell fractions were enriched in CSCs, we quantified octamer-binding transcription factor-4 (Oct-4) and sry-related HMG box-containing (Sox2) mRNA levels in cells by RT-qPCR. Oct-4 and Sox2 expression levels were significantly higher in CD133^+^ cell fractions than in CD133^−^ cell fractions (Fig 1B). These results confirmed that the CD133^+^ subpopulation displayed CSCs features, consistent with previous studies [18].In this study, we designated CD133^+^ cell fractions as pancreatic cancer stem-like cells whereas CD133^−^ cell fraction utilized as non-stem cells.

To determine if pancreatic cancer stem-like cells were a suitable model for CCR7-mediated potentiality of CCL21-driven pancreatic carcinoma metastasis, we evaluated chemokine receptor expression levels in total, CD133^+^, and CD133^−^ pancreatic cancer cells by RT-qPCR. CCR7 mRNA was preferentially expressed in CD133^+^ cell fractions, low expressed in parental cell line and almost un-expressed in CD133^−^ fractions (Fig 1C). Immunofluorescence analysis revealed similar results; CCR7 expression was increased in CD133^+^ cell fractions but very low in CD133^−^ fractions (Fig 1B). Additionally, similar data were also obtained from AsPC-1 and MIA PaCa-2 cells lines (S1 Fig).

### CCL21/CCR7 increases the migration potential of CD133^+^ pancreatic cancer stem-like cells *in vitro*

**Fig 2:**
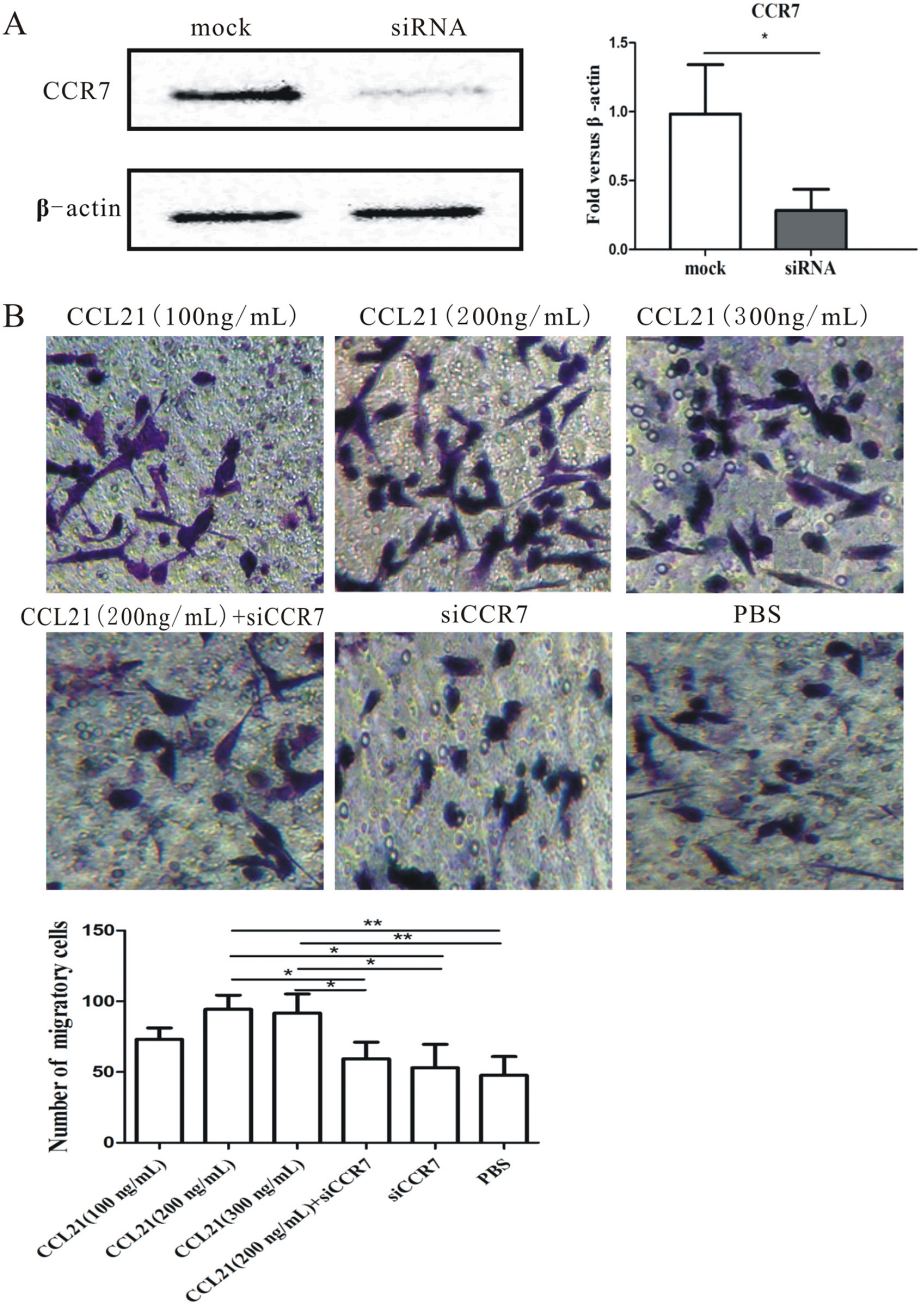
Effect of CCL21/CCR7 on migration of CD133^+^ pancreatic cancer stem-like cells *in vitro*. (A) CD133^+^ cells were transfected with non-targeted control siRNA and siRNA directed against CCR7. CCR7 levels were analyzed 48h after transfection by western blot. Data were normalized to β-actin levels. Experiments were repeated three times with similar results. (B) CD133^+^ cell migration was analyzed by Boyden chamber migration assays. CD133^+^ cells were treated for 24h with different concentrations of CCL21 (a, 100 ng/mL; b, 200 ng/mL; c, 300 ng/mL), CCL21(200 ng/mL) +siCCR7(d), siCCR7(e), and PBS(f), respectively. Cells that migrated to the lower chamber were fixed, stained, and counted. Migratory cells were counted in at least three to four randomly-selected microscopic fields and the results are expressed as the mean ± standard deviation (SD) of migratory cells per microscopic field. Experiments were repeated three times and the data were expressed as mean± SD. The difference between these two cell populations was significant(*P<0.05, **P<0.01, ***P<0.001).

We tested the hypothesis that CCL21/CCR7 increases the migration potentiality of pancreatic cancer stem-like cells as well as promoting survival, by CCR7 knockdown with small interfering (si) RNA. Western blot confirmed significant, specific, and sustained down-regulation of CCR7 following siCCR7 treatment in CD133^+^ cell fractions, whereas CCR7 expression levels remained unchanged in mock-transfected control CD133^+^ cell fractions (Fig 2A).

We investigated the role of CCL21/CCR7 in regulating the migratory ability of CD133^+^ cell fractions by Transwell assays. CCR7 activation and inhibition were induced by exogenous CCL21 and siCCR7, respectively. After cell sorting, CD133^+^ cell fractions were plated in the upper chamber for 2h and treated with the following agents for 24h: (1) phosphate-buffered saline (PBS), (2) CCL21 (100, 200, and 300 ng/mL, respectively), (3) CCL21 (200 ng/mL) + siCCR7, and (4) siCCR7. As Fig 2B shown, 200 ng/mL or 300 ng/mL CCL21 both significantly promoted cell migration comparing with control, with peak concentration on 200 ng/mL; although 100 ng/mL CCL21 also contributed to the cell migration; there was no statistical significance. Additionally, similar data were also obtained from AsPC-1 and MIA PaCa-2 cell lines (S2 Fig). These results suggest that **C**CL21/CCR7 increased the migration potential of CD133^+^ pancreatic cancer stem-like cells *in vitro*.

### CCL21/CCR7 promotes survival in starved CD133^+^ pancreatic cancer stem-like cells *in vitro*

**Fig 3:**
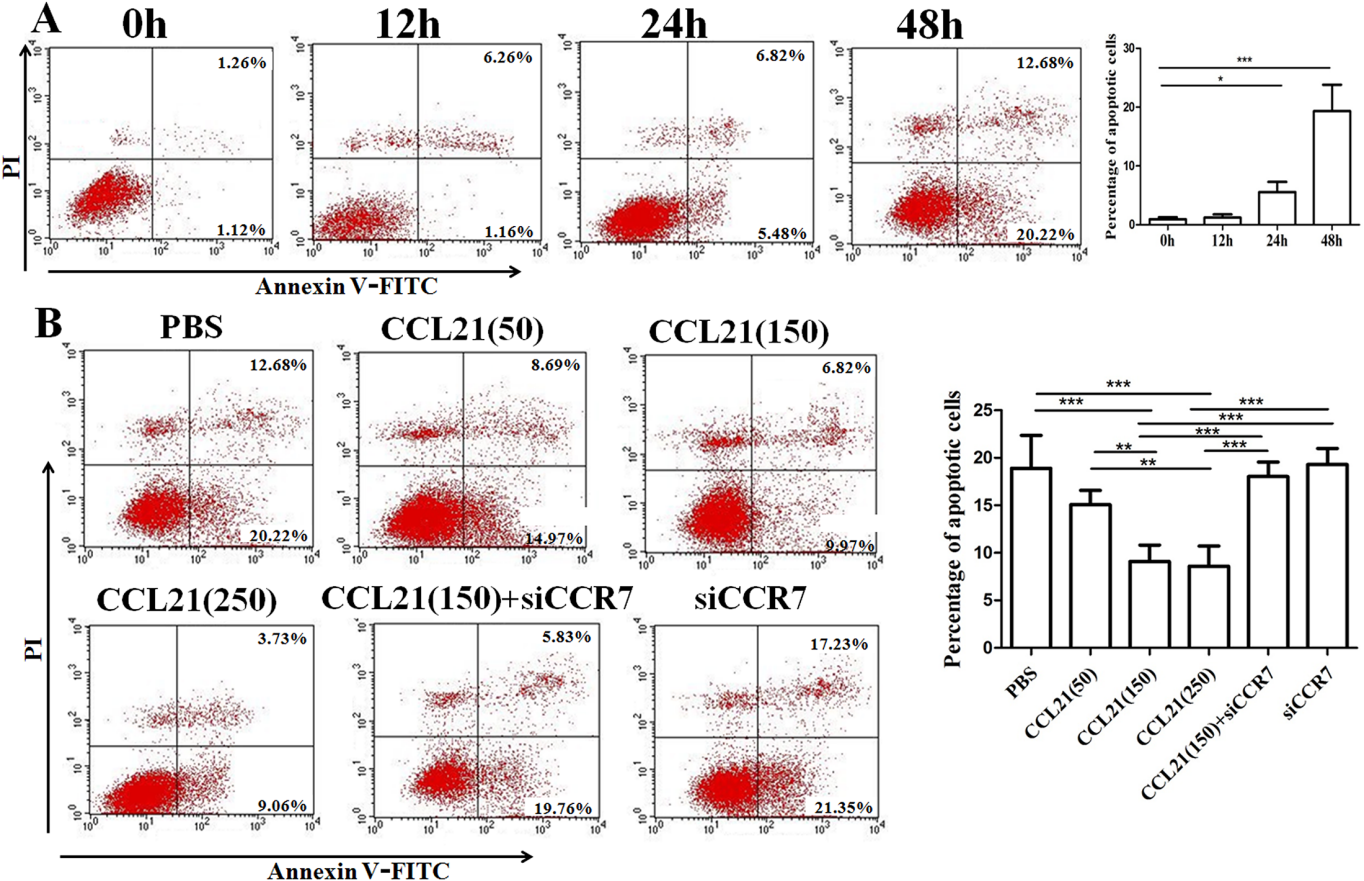
Effect of CCL21/CCR7 on survival of CD133^+^ pancreatic cancer stem-like cells *in vitro*. (A) HBSS starvation induced CD133^+^ apoptosis in a time-dependent manner. Cells were cultured in HBSS for 0, 12, 24, or 48 h followed by flow cytometry detection of phosphatidylserine exposure analyzed by Annexin V and propidium iodide (PI) staining. The apoptotic cells have been clearly increased in a time dependent manner. (B) Exogenous CCL21 protein promoted CD133^+^ cell survival under HBSS conditions. Cells were treated for 48 h with different concentrations of CCL21 (a, 50ng/mL; b, 150ng/mL; c, 250 ng/mL), CCL21 (150 ng/mL)+ siCCR7(d), siCCR7(e), and PBS(f). Experiments were repeated three times and the data were expressed as mean± SD. The difference between these two cell populations was significant (*P<0.05, **P<0.01, ***P<0.001).

Survival of CTCs in the bloodstream is essential for tumor cell metastasis. We examined the ability of CCL21/CCR7 to promote CD133^+^ pancreatic cancer stem-like cells survival. CD133^+^ cell fractions were cultured in hank’s balanced salt solution (HBSS) for 0, 12, 24, and 48 h [19].As shown in Fig 3A, HBSS starvation significantly induced apoptosis in CD133^+^ cells in a time-dependent manner. To evaluate the effect of CCL21/CCR7 on CD133^+^ cell survival, CD133^+^cell apoptosis was pre-treated by the following agents: (1) PBS, (2) CCL21 (50, 150, and 250 ng/mL, respectively), (3) CCL21 (150 ng/mL) + siCCR7, and (4) siCCR7 and then cultured in HBSS for 48 h. The results showed that proportion of apoptotic cells were significantly reduced following the CCL21 treatment, especially at a concentration of 150 ng/mL CCL21, and this effect was reversed by siCCR7(Fig 3B).

### CCL21/CCR7 promotes EMT in CD133^+^ pancreatic cancer stem-like cells *in vitro*

**Fig 4:**
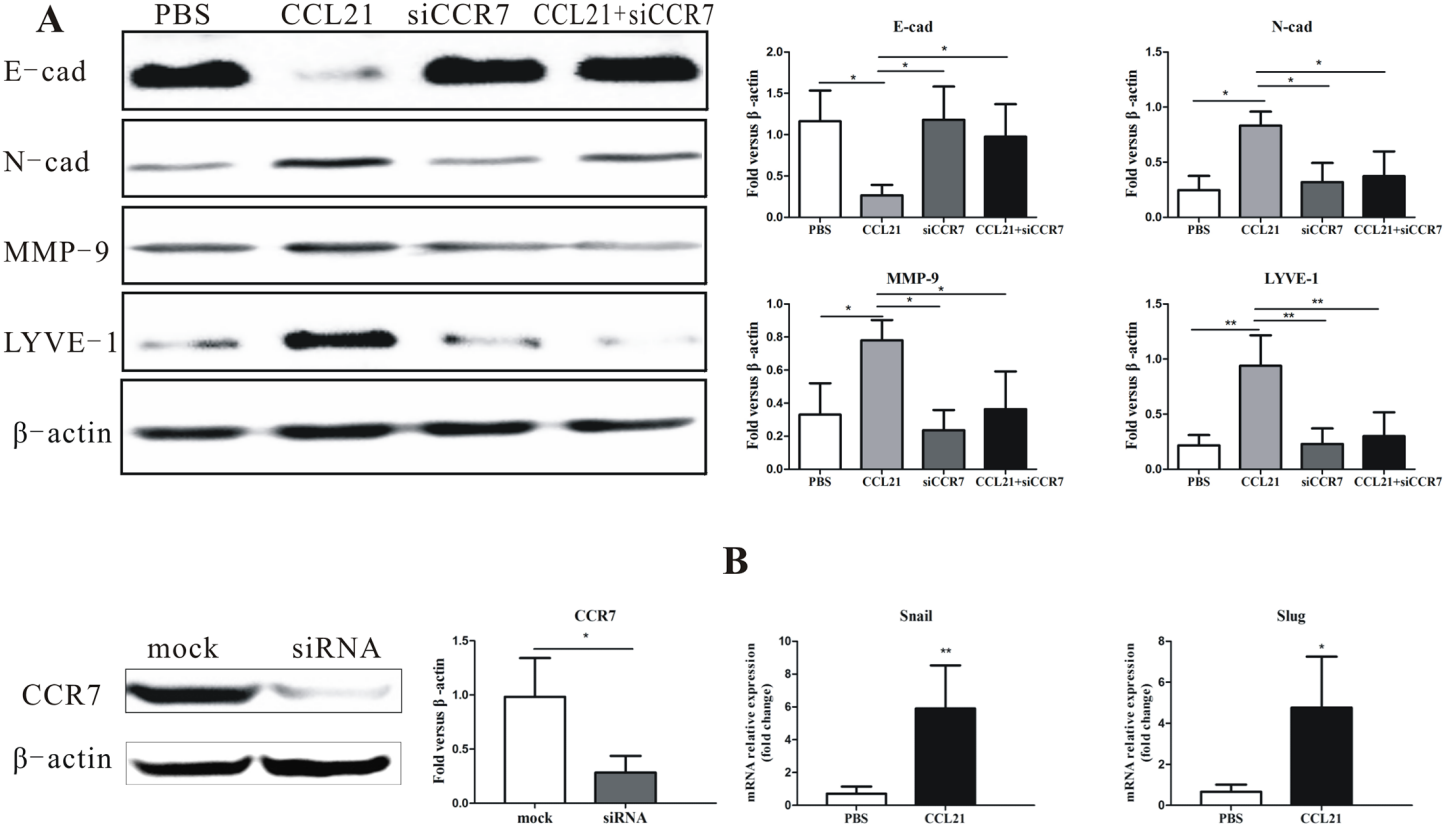
Effect of CCL21/CCR7 on EMT in pancreatic cancer stem cells. (A) Expression of E-cad, N-cad, MMP-9, and LYVE-1 proteins in CD133^+^ cells detected by western blot during treatment with CCL21(200 ng/mL), CCL21(200 ng/mL)+ siCCR7, siCCR7, and PBS. CD133^+^ cells were transfected with non-targeted control siRNA and siRNA directed against CCR7. CCR7 levels were analyzed 48h after transfection by western blot. Data were normalized to β-actin levels. Experiments were repeated three times with similar results. (B) Expression levels of Snail and Slug were measured by RT-qPCR after treatment of CD133^+^ with CCL21 (200 ng/mL) or PBS. Data were normalized to β-actin levels. Experiments were repeated three times and the data were expressed as mean± SD. The difference between these two cell populations was significant (*P<0.05, **P<0.01, ***P<0.001).

As shown above, peak migration was induced by 200 ng/mL CCL21. We therefore used CCL21 at the concentration of 200 ng/mL in the following experiments. Numerous studies have demonstrated that EMT is required for tumor metastasis [8–11]. We investigated the role of CCL21/CCR7 in EMT and lymph node metastasis by analyzing their effects on E-cadherin (E-cad), N-cadherin (N-cad), matrix metalloproteinase-9 (MMP-9), and lymphatic vessel endothelial hyaluronan receptor E-1(LYVE-1) by western blot. We treated CD133^+^ cells with the following agents for 24h: (1) PBS, (2) CCL21 (200 ng/mL), (3) CCL21 (200ng/mL) + siCCR7, and (4) siCCR7. CCL21 enhanced the expression of N-cad and MMP-9 and inhibited the expression of E-cad compared with the control group, indicating a shift from epithelial to mesenchymal phenotype (Fig 4A). Given that CCL21/CCR7 prompted EMT, then Snail and Slug, the EMT-inducing transcription factors, were detected by RT-qPCR. Snail and Slug mRNA were up-regulated by CCL21 (Fig 4B). CCL21 also enhanced the expression of the specific lymph node metastasis marker LYVE-1(Fig 4A).

These results suggest that CCL21/CCR7 regulated metastasis of pancreatic carcinoma cells, including lymph node metastasis, by modulating the expression levels of cadherin and EMT transcription factors.

### CCL21/CCR7 induces activation of the Erk/ NF-κB pathway in CD133^+^ pancreatic cancer stem-like cells

Extracellular-signal regulated kinase (Erk) and downstream regulators of nuclear factor (NF)-κB have been implicated in cancer cell migration and metastasis. We therefore evaluated the effect of CCL21/CCR7 on Erk and NF-κB activation in CD133^+^ pancreatic cancer stem-like cells.

**Fig 5:**
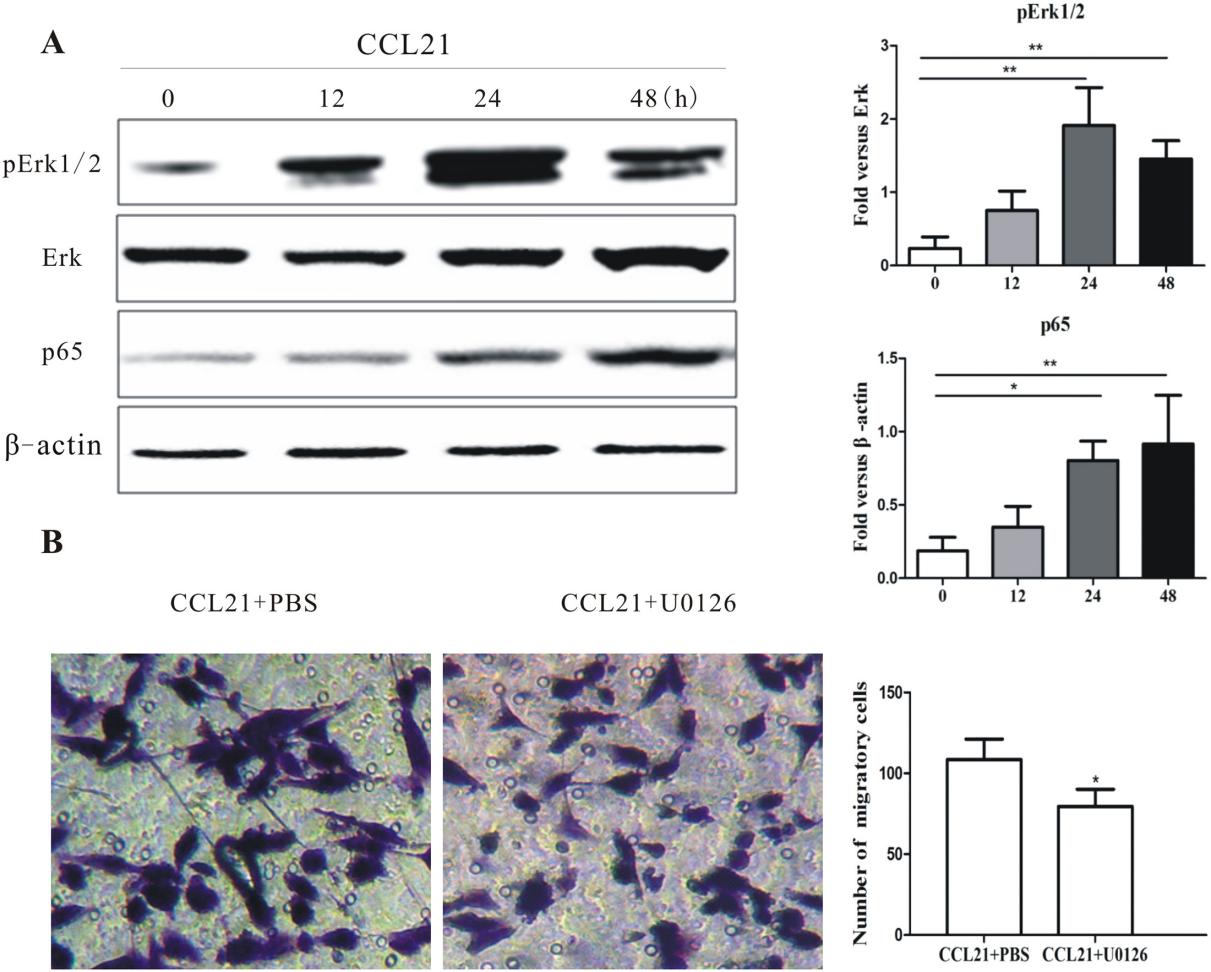
Effect of CCL21/CCR7 on Erk/NF-κB activation in pancreatic cancer stem cells. (A) Treatment of CD133^+^ cells with CCL21 induced pErk1/2 and p65 activation in time-dependent manners. CD133^+^ cells were treated with CCL21 (200 ng/mL) for different durations (0, 12, 24, or 48 h) and then harvested. Cell lysates were subjected to western blot analysis. Data were normalized to β-actin levels. (B) Treatment of CD133^+^ cells with an Erk1/2 inhibitor (U0126) inhibited cell migration compared with control group. Experiments were repeated three times and the data were expressed as mean± SD. The difference between these two cell population was significant (*P<0.05, **P<0.01, ***P<0.001).

Following the CCL21 treatment, Erk1/2 in CD133^+^ cells was phoshporylated in a time-dependent manner (Fig 5A). Similarly, CCL21 also promoted p65, a subunit of NF-κB, expression in CD133^+^ cells. To confirm Erk1/2 signaling was involved in CCL21/CCR7 induced CD133^+^ cell migration, U0126, an inhibitor of Erk1/2 was employed. CD133^+^ cells were pretreated with U0126 (10μmol/L) or PBS (control) for 1 h followed by CCL21 (200 ng/mL) for 48h. Cell migration was significantly inhibited (Fig 5B).

### CCR7 expression in pancreatic cancer and adjacent normal tissues

**Fig 6:**
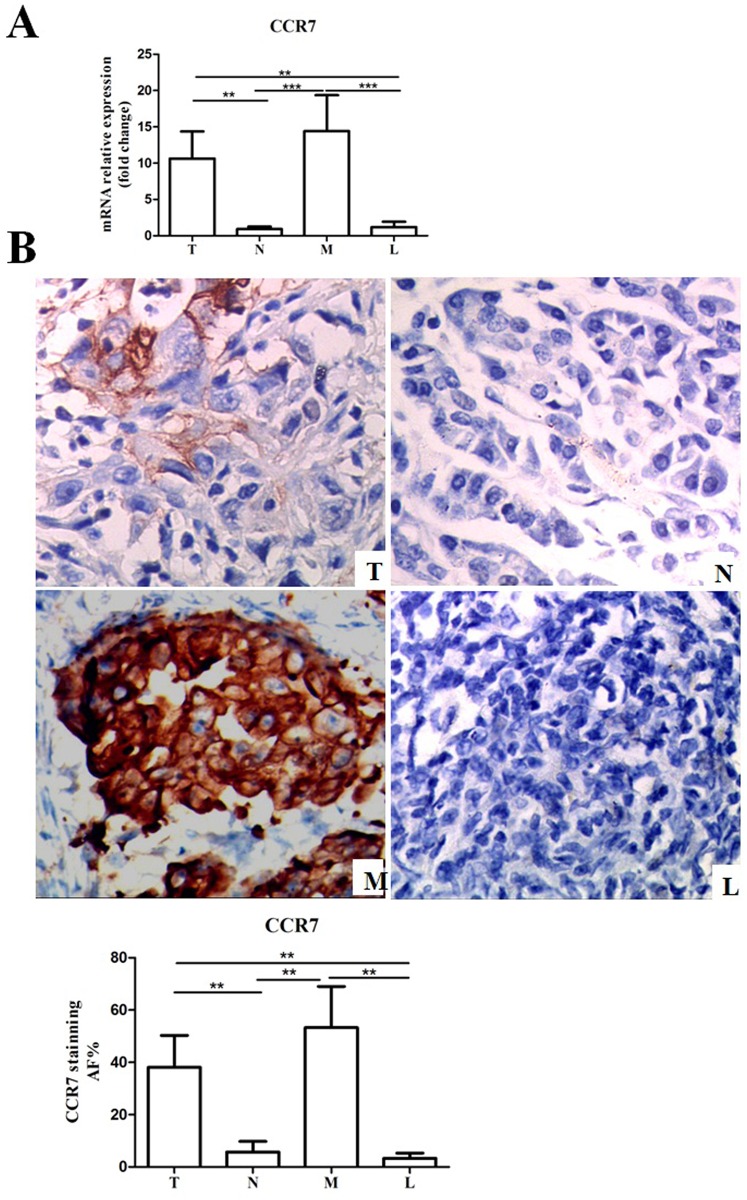
CCL21/CCR7 expression was up-regulated in pancreatic carcinoma patients. (A) CCR7 mRNA expression levels in resected pancreatic cancer tissues, metastatic lymph nodes, normal lymph nodes and adjacent normal tissues were determined using RT-qPCR. Data were normalized to β-actin expression. (B) CCR7 expression levels were detected in normal lymph nodes, adjacent normal tissues, resected pancreatic cancer tissues, and metastatic lymph nodes by immunohistochemistry(400×). All experiments were repeated three times (*P<0.05, **P<0.01, ***P<0.001). T, resected pancreatic cancer tissue; M, metastatic lymph node; N, adjacent normal tissue; L, normal lymph node.

Previous studies have suggested that CCL21 is highly expressed in some solid cancers with lymph node metastasis [16]. We therefore detected the presence of CCR7 in resected pancreatic cancer tissues, metastatic lymph nodes, normal lymph nodes, adjacent normal tissues. CCR7 expression was up-regulated in resected pancreatic cancer tissues and metastatic lymph nodes, especially, in metastatic lymph nodes (15 folds) compared with adjacent normal tissues and normal lymph nodes (Fig 6A). CCR7 protein levels were detected in normal lymph nodes, adjacent normal tissues, resected pancreatic cancer tissues, and metastatic lymph nodes by immunohistochemistry. CCR7 protein levels were increased in resected pancreatic cancer tissues and metastatic lymph nodes compared with adjacent normal tissues and normal lymph node (Fig 6B). These results suggest that CCL21/CCR7 up-regulation might be associated with lymph node metastasis in patients with pancreatic carcinoma.

## Materials and Methods

### Patients and samples

Forty patients (20 women and 20 men; mean age 58 years) with pancreatic carcinoma who were operated on at the Affiliated Hospital of Jiangsu University from 2009 to 2012 were enrolled in this study. The resected specimens (primary tumor tissues and lymph node tissues) were fixed in 10% neutral buffered formalin and embedded in paraffin. Information on patient age, sex, tumor location, tumor size and status of lymph node metastasis were obtained from histopathology records. The clinic pathological characteristic of the 40 pancreatic carcinoma patients was summarized in Table 1.Written informed consent was obtained from each patient. The study protocol was approved by the Ethics Committee of the Affiliated Hospital of Jiangsu University. None of the patients received radiotherapy, chemotherapy or other medical interventions before the study.

**Table 1:** Clinic pathological and biological characteristics of the pancreatic carcinoma patient profiles (n = 40).

Variable	Frequency (%)
**Age(years)**	
<50	14(35%)
≥50	26(55%)
**Sex**	
Male	25(63%)
Female	15(37%)
**Tumor location**	
Head of pancreatic	22(55%)
Body of pancreatic	11(28%)
Tail of pancreatic	7(17%)
**Tumor size**	
<2cm	16(40%)
≥2cm	24(60%)
**Lymph node metastasis**	
regional	12(30%)
distant	13(33%)

### Cell culture

Panc-1, AsPC-1 and MIA PaCa-2 pancreatic cancer cell lines were obtained from the Cell Bank of the China Academy of Sciences (Shanghai, China). Cells were maintained in DMEM-F12 medium supplemented with 10% fetal bovine serum (FBS, Gibco, NY, USA), 100 U/mL penicillin, and 100 U/mL streptomycin. The cancer stem-like cell fraction was propagated using culture conditions favoring proliferation of undifferentiated cells. Cells were cultured in serum-free DMEM-F12 medium containing insulin (Gibco), epidermal growth factor (EGF), basic fibroblast growth factor (bFGF, Preprotech, Rocky Hill, NJ, USA), and B-27 (Gibco), in low-attachment dishes (Corning, NY, USA). Cells were passaged with 0.25% trypsin/EDTA every 3 days. HBSS was purchased from Invitrogen (Invitrogen, Shanghai, China) and buffered with 2.2 g/L NaHCO_3_ (Merck, Shanghai, China).

### Transwell assay

Cell migration assays were performed in Transwell chambers using 8-μm pore size filters (Corning, NY, USA). Briefly, a total of 1×10^4^ viable cells were plated in the upper chamber in serum-free medium. Two hours later, the cells were treated simultaneously with CCL21 (Preprotech, 100, 200, or 300 ng/mL), siCCR7, or PBS (control). After 24 h incubation at 37°C, 95% air, 5% CO_2_ atmosphere, cells in the upper chamber and on the upper filter surface were removed, whereas cells on the lower filter surface were fixed with ethanol and stained with Giemsa. The number of migrating cells was determined by counting cells in 10 random fields at ×200 magnification. All experiments were performed in triplicate.

### siRNA and transfection

Cells (10^5^ cells/well) were cultured overnight in six-well plates. Following normalization, cells were transfected with 50 nmol/L negative control siRNA or CCR7-specific siRNA (Suzhou Ribo Life Science Co., Ltd., Suzhou, China) according to the manufacturer’s instructions. Protein lysates were collected at 48h and levels of CCR7 protein were assessed by western blot. The sequences of the siCCR7 and control siRNAs were as follows: CCR7,5′-GCGUCAACCCUUUCUUGUATT-3′ and 3′-UACAAGAAAGGGUUGACGCAG-5′; and control,5′-UUCUCCGAACGUGUCACGUTT-3′ and 3′-ACGUGACACGUUCGGAGAATT-5′.

### Apoptosis analysis

The apoptotic ratios of cancer cells were determined using Annexin V-FITC/propidium iodide (PI) apoptosis detection kits (Invitrogen, Shanghai, China).Apoptotic cells were analyzed based on PI and Annexin V staining. Briefly, after treatment with different concentrations of CCL21, the cells were collected and washed twice with ice-cold PBS buffer, re-suspended in 100μL of 1×Annexin binding buffer, incubated with 5 μL of Annexin V conjugated to FITC and 1 μL PI (100 mg/mL) for 15 min at room temperature, then resuspended in 400 μL of 1×Annexin binding buffer and analyzed by flow cytometry.

### Western blot analysis

Protein concentrations were determined using the bicinchoninic acid method. Cell lysates were subjected to sodium dodecyl sulfate-polyacrylamide gel electrophoresis and transferred to polyvinylidene fluoride membranes (Merck Millipore, MA, USA). Membranes were blocked with 5% (w/v) bovine serum albumin (BSA) in TBST for 1 h at room temperature and incubated overnight with primary antibodies at 4°C. They were subsequently incubated with horseradish peroxidase-conjugated second antibodies for 2 h at room temperature. The results were normalized to β-actin as appropriate. The immunoreactive bands were detected by chemiluminescence (ECL Plus, Merck Millipore) and relevant blots were quantified by densitometry using LANE-1D software. Primary antibodies were as follows: rabbit-anti-human-E-cad, N-cad, MMP-9, Erk, pErk1/2 and p65. All antibodies were obtained from Cell Signaling Technology, Inc. (Boston, MA, USA). Anti-β-actin, rabbit-anti-human-CCR7 and LYVE-1 were obtained from Abcam Company (Cambridge, MA, USA). Secondary antibody preparations were either anti-rabbit or anti-mouse were purchased from Boster Biotechnology Company (Wuhan, China).

### RT-qPCR

RT-qPCR was carried out with SYBR Green qPCR SuperMix (Bio-Rad, Hercules, CA, USA) using the CFX-96 system (Bio-Rad). Total cellular and tissue RNA was isolated using TRIzol reagent, and cDNA was synthesized from 1 μg of total RNA using oligo dT and murine Moloney leukemia virus reverse transcriptase (Toyobo, Osaka, Japan). Relative expression levels of the genes were calculated using the 2^−ΔΔCT^ method. The following gene-specific primers were used: Oct-4, 5′-GGACCAGTGTCCTTTCCTCT-3′ and 5′-CCAGGTTTTCTTTCCCTAGC-3′; Sox2, 5′-ACCCCAAGATGCACAACTC-3′ and 5′-GCTTAGCCTCGTCGATGAAC-3′; Snail, 5′-ACCCCACATCCTTCTCACTG-3′ and 5′-TACAAAAACCCACGCAGACA-3′; Slug,5′-ACACACACACACCCACAGAG-3′ and 5′-AAATGATTTGGCAGCAATGT-3′; and CCR7, 5′-TGAGGTCACGGACGATTACAT-3′ and 5′-GTAGGCCCACGAAACAAATGAT-3′.

### Flow cytometry analysis

Cells (5×10^6^) were harvested, disaggregated into single-cell suspensions, and stained with 1.25 μg/ml mouse anti-human phycoerythrin (PE)-labeled CD133 (clone AC133, Miltenyi Biotec. Company, CA, USA). The antibody was incubated for 30 min at 4°C in the dark. After incubation, the samples were washed with PBS and analyzed by FACS AriaII (Becton Dickinson, USA). CD133^+^ pancreatic cancer cells were sorted and selected for experiments. The sorting gate was established using cells stained with isotype control PE-conjugated mouse IgG1 antibody (Miltenyi Biotec. Company).

### Immunofluorescence staining

CD133^+^ and CD133^−^ cells were seeded in 24-well plates. After 10 h, medium was aspirated from the plates and the cells were fixed with 4% paraformaldehyde solubilized in PBS/0.1%Triton-X100 for 30 min at room temperature, then blocked for 1 h with PBS/1%BSA Triton-X100. The primary antibody was applied for 2 h at room temperature. After washing, the cy3-labeled second antibody was added for 90 min. Finally, the cells were stained with Hoechst 33342 for 10 min.

### Immunohistochemical staining

Tissues were cut into 4μm slices, incubated at 60°C for 30 min, and dewaxed in xylene. After dewaxing and antigen unmasking, endogenous peroxidases were blocked with 90% methanol/3% H_2_O_2_ for 15 min at room temperature. The slides were then blocked in blocking buffer for 20 min. Primary antibodies were applied overnight at 4°C followed by washing and addition of biotin-labeled second antibody for 20 min. Strept avidin-biotin complex (SABC) was then added for 20 min. Finally, DAB was added immediately to the slides to produce the color change. All the sections were cover slipped with neutral balsam and viewed under an Olympus microscope and analyzed using Image J software.

### Statistical analysis

Differences between groups were analyzed using Student’s *t*-tests or one-way ANOVA. Values of P<0.05 were considered to be significant. All experiments were performed at least in triplicate.

## Discussion

Pancreatic carcinoma remains a devastating disease. Most pancreatic cancer-associated deaths are attributed to metastasis of the primary tumor, rather than development of the primary tumor itself, though our understanding of this complex problem remains limited.

Tumor metastasis is driven not only by the accumulation of intrinsic alterations in malignant cells, but also by interactions between cells and diverse components of the tumor microenvironment [20, 21]. Successful metastatic outgrowth thus depends on the ability of cancer cells to take advantage of the surrounding microenvironment at each step of the metastatic process. Recently, more and more studies have provided evidence of self-renewing, stem-like cells within tumors, namely CSCs. Until now, CSCs have been discovered in several solid tumors including pancreatic carcinoma. CSCs play a crucial role in tumor initiation, maintenance, chemo- or radio- resistance and metastasis. The surface marker CD133 was reported to have the properties of CSCs in pancreatic carcinoma. According to the former reports, we designated CD133 positive cells as pancreatic cancer stem-like cells whereas CD133 negative cells utilized as non-stem cells in this study[22, 23]. Oct-4 and Sox2 are core regulators in stem cell self-renew and validated as cancer stem cell target [24, 25]. After sorting CD133 positive cells, we further analyzed Oct-4 and Sox2 mRNA expression levels in the CD133 positive cells to confirm whether these subpopulation acquisition of a stem cell phenotype.

Tumor cell migration and metastasis, which share many similarities with leukocyte trafficking, are critically regulated by chemokines and their receptors. Accumulating evidence suggests that over-expression of CCR7 is correlated with lymphatic invasion; CCL21/CCR7 regulated migration and metastasis in a variety of lung, esophageal, and pancreatic cancer cells, possibly allowing them to access the lymphatic system and spread to regional lymph nodes[14–16, 26–28]. The results of the present study detected high levels of CCL21/CCR7 in pancreatic carcinoma tissues and metastasis lymph nodes. Consistent with former research, CCL21/CCR7 was highly expressed in cancer tissues, especially in patients with lymph node metastasis. Furthermore, our *in vitro* results demonstrated that pancreatic CSCs expressed high levels of CCR7, suggesting that CSCs may represent a subpopulation capable of initiating metastasis[29]. However, our understanding of these complex connections between CCL21/CCR7, CSCs, and metastasis remains limited.

EMT is a process through which epithelial cells lose their epithelial traits and acquire instead the attributes of mesenchymal cells, with loss of E-cad and increased expression of N-cad and vimentin. Transcription factors, such as Snail, Slug and Twist have been shown to act as vital controller of the EMT [11].An emerging concept for metastasis suggests that cellular plasticity associated with EMT is critical for the ability of cancer cells to disseminate from the primary tumor site and survive circulation, and for their enhanced migratory capacity, invasiveness, and increased resistance to apoptosis. Indeed, a population of pancreatic cells that exhibited EMT was shown to be locally invasive and cause the introduction of CTCs into the blood stream before frank malignancy could be observed[30, 31]. In addition to cell detachment and increased migratory capacity, EMT has also been correlated with the acquisition of stemness properties, which contribute to metastatic ability [11]. The results of our study extended the analysis of EMT related markers in pancreatic CSCs after treatment with CCL21; treatment of pancreatic CSCs with CCL21 resulted in promotion of EMT related markers and transcription factors, as well as promotion of survival, which effects were inhibited by siCCR7. The hyaluronan receptor LYVE-1 has been widely used for the detection of tumor-associated lymphatic vessels in different types of tumors. An increased LYVE-1 protein level is closely associated with key adverse risk factors and lymph node metastasis [32]. Our study found that the expression level of LYVE-1 increased in pancreatic cancer stem cells after treatment with CCL21, which supply the direct molecular mechanism that CCL21 was responsible for mediating lymph node metastasis.

Pancreatic cancer cells are known to overexpress NF-κB[33]. Several studies in other cell types have indicated that activation of CCR7 is associated with increased phosphorylation of Erk, which is an upstream regulator of NF-κB[34–36]. Erk/NF-κB is known to regulate a wide spectrum of cancer properties, including cell proliferation and anti-apoptosis, and also to play critical roles in cell migration and metastasis. Importantly, NF-κB has recently been identified as an important regulator of EMT in many cancer cell types [37, 38]. CCL21/CCR7 up-regulated the levels of Erk/NF-κB in pancreatic CSCs and may help to promote their migratory capacity. This hypothesis is further supported by the fact that pancreatic CSC migration was reduced by treatment with the Erk1/2-specific inhibitor UO126.

## Conclusions

The results of this study provide the evidence demonstrating that CCL21/CCR7 promotes migration and survival of pancreatic CSCs by activating Erk/NF-κB signaling and promoting EMT. However, more studies are needed to identify and evaluate the direct molecular mechanisms responsible for these processes. Further insights into these mechanisms may provide novel targets for the prevention and treatment of pancreatic cancer metastasis.

## Supporting Information

S1 Fig Expression levels of CCR7 in AsPC-1 and MIA PaCa-2. CD133^+^ and CD133^−^ cells were sorted from total AsPC-1 and MIA PaCa-2 cells lines by FACS. CCR7 expression levels in total pancreatic cancer cells and in CD133^+^ and CD133^−^ cell fractions were detected by immunofluorescence staining (200×)(**P<0.01, ***P<0.001).
(TIF)

S2 Fig Effect of CCL21/CCR7 on migration of CD133^+^ pancreatic cancer stem-like cells from AsPC-1 and MIA PaCa-2 *in vitro*. The migration ability of CD133^+^ cell fom AsPC-1(A) and MIA PaCa-2(B) was analyzed by Boyden chamber migration assays. CD133^+^ cells were treated for 24h with 200 ng/mL CCL21, CCL21 (200 ng/mL) +siCCR7, siCCR7, and PBS, respectively. Cells that migrated to the lower chamber were fixed, stained, and counted. Migratory cells were counted in at least three to four randomly-selected microscopic fields and the results are expressed as the mean ± standard deviation (SD) of migratory cells per microscopic field. Experiments were repeated three times and the data were expressed as mean± SD. The difference between these two cell populations was significant (*P<0.05, **P<0.01, ***P<0.001).
(TIF)

## Abbreviations

bFGF: basic fibroblast growth factor
CCL21: chemokine ligand 21
CCR7: C-C chemokine receptor 7
CD133: cluster of differentiation 133
CSCs: cancer stem cells
EGF: epidermal growth factor
E-cad: E-cadherin
EMT: epithelial-to-mesenchymal transition
Erk: extracellular-signal regulated kinase
HBSS: Hank's balanced salt solution
LYVE-1: lymphatic vessel endothelial hyaluronan receptor-1
MMP-9: matrix metalloproteinases-9
N-cad: N-cadherin
OCT-4: octamer-binding transcription factor-4
PBS: Phosphate Buffered Saline
RT-qPCR: real time- quantitative polymerase chain reaction
SABC: strept avidin-biotin complex
Sox2: sry-related HMG box-containing
