# Supplementary figures and images for: CCL21/CCR7 Axis Contributed to CD133+ Pancreatic Cancer Stem-Like Cell Metastasis via EMT and Erk/NF-κB Pathway
Source: PLoS One. 2016 Aug 9;11(8):e0158529. doi: 10.1371/journal.pone.0158529 (PMC4978474; doi:10.1371/journal.pone.0158529)

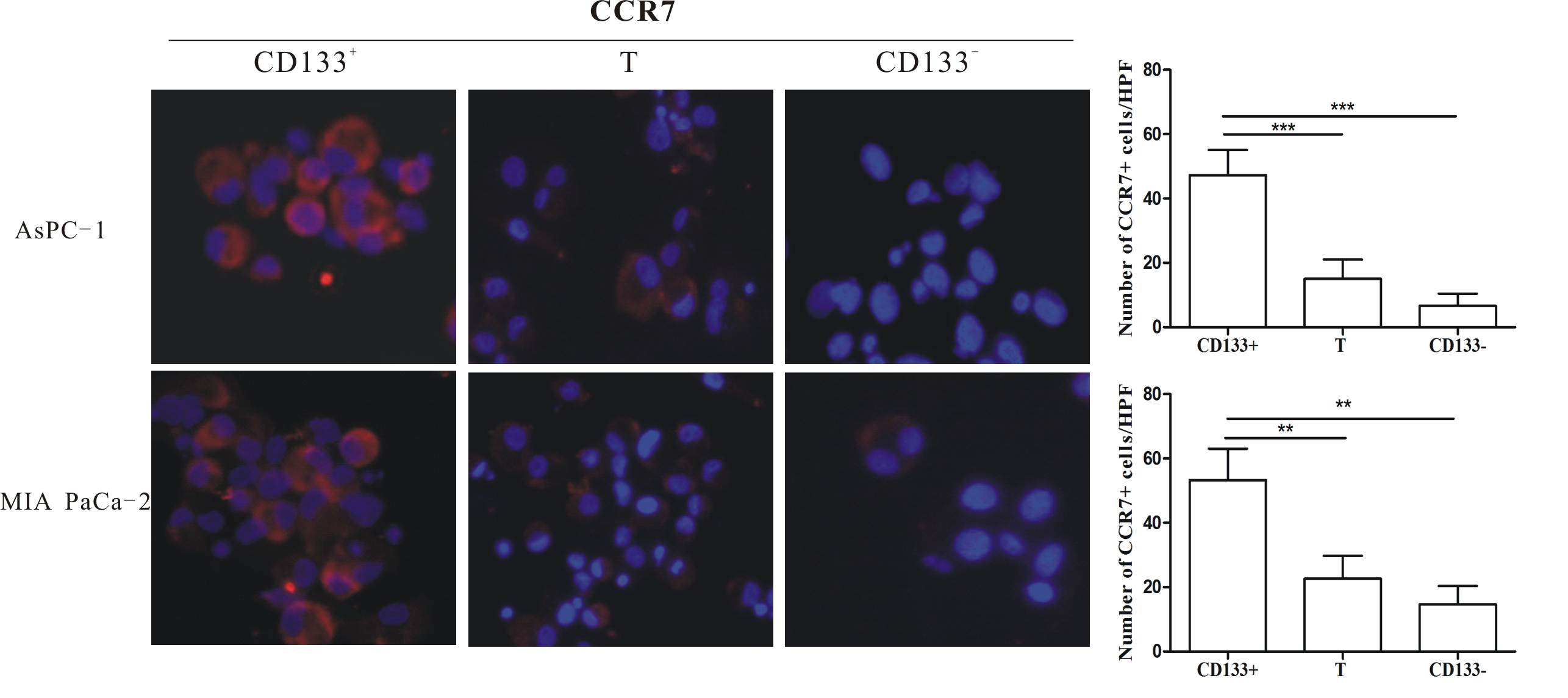

Supplement: S1 Fig — CD133+ and CD133− cells were sorted from total AsPC-1 and MIA PaCa-2 cells lines by FACS. CCR7 expression levels in total pancreatic cancer cells and in CD133+ and CD133− cell fractions were detected by immunofluorescence staining (200×)(**P<0.01, ***P<0.001). (TIF) [file pone.0158529.s001.tif]

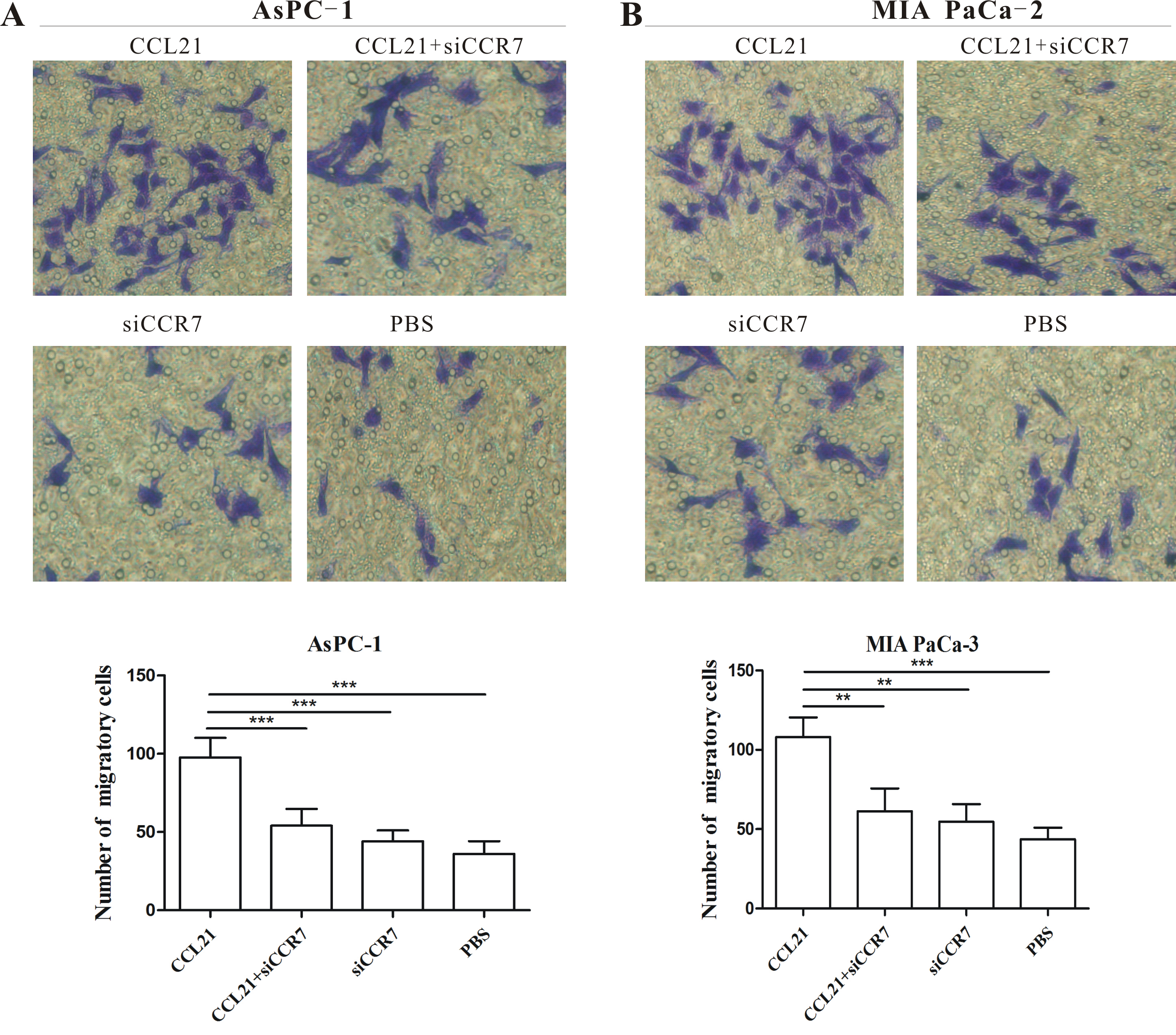

Supplement: S2 Fig — The migration ability of CD133+ cell fom AsPC-1(A) and MIA PaCa-2(B) was analyzed by Boyden chamber migration assays. CD133+ cells were treated for 24h with 200 ng/mL CCL21, CCL21 (200 ng/mL) +siCCR7, siCCR7, and PBS, respectively. Cells that migrated to the lower chamber were fixed, stained, and counted. Migratory cells were counted in at least three to four randomly-selected microscopic fields and the results are expressed as the mean ± standard deviation (SD) of migratory cells per microscopic field. Experiments were repeated three times and the data were expressed as mean± SD. The difference between these two cell populations was significant (*P<0.05, **P<0.01, ***P<0.001). (TIF) [file pone.0158529.s002.TIF]
